# A Case of Achalasia and Aspiration Pneumonia in a Young Adult

**DOI:** 10.7759/cureus.111211

**Published:** 2026-06-20

**Authors:** Garrett Perchetti, Katherine Becker, Julia Speiser, Saketh Jayanthi, David Mushayev, Roxana Lazarescu

**Affiliations:** 1 Internal Medicine, Wyckoff Heights Medical Center, New York, USA; 2 Internal Medicine, Touro College of Osteopathic Medicine, New York, USA

**Keywords:** achalasia management audit, acute asthma, esophageal achalasia, gastroesophageal reflux disease (gerd), motor dysfunction of the esophagus, severe achalasia

## Abstract

Achalasia is an uncommon primary esophageal motility disorder characterized by impaired lower esophageal sphincter relaxation and absent esophageal peristalsis. These changes result in progressive esophageal dilation and functional obstruction. Although classically associated with dysphagia and regurgitation, respiratory manifestations may predominate due to chronic aspiration of retained esophageal contents, which can obscure the underlying diagnosis and delay appropriate management. We report the case of a 20-year-old male with a history of asthma, gastroesophageal reflux disease, and gastroparesis who presented with one day of shortness of breath and productive cough, initially treated as an asthma exacerbation. Imaging performed to evaluate new-onset respiratory symptoms for one day prior to admission revealed a markedly dilated, fluid-filled esophagus and radiographic findings consistent with aspiration pneumonia. He was initially treated with one dose of 2 mg IV cefepime, then modified to Unasyn 3 mg and doxycycline 100 mg. Subsequent endoscopic evaluation confirmed severe esophageal dilation with retained food contents, supporting the diagnosis of advanced achalasia. This case highlights how achalasia may present primarily with pulmonary complications in young patients, particularly when coexisting respiratory disease is present. Delayed recognition can result in significant morbidity, including recurrent aspiration, malnutrition, and progressive esophageal dysfunction. Early consideration of esophageal motility disorders in patients with refractory respiratory symptoms and GI history is essential to prevent complications and guide timely therapeutic intervention.

## Introduction

Achalasia is a rare primary esophageal motility disorder characterized by impaired relaxation of the lower esophageal sphincter (LES) and loss of coordinated peristalsis within the esophageal body. The disorder results from degeneration of inhibitory neurons within the myenteric plexus, producing functional obstruction at the esophagogastric junction and progressive esophageal dilation over time [1,2]. Although achalasia can occur within any age group, sex, or ethnicity, diagnosis is frequently delayed due to its gradual onset and symptom overlap with more prevalent GI disorders. It is most often associated with male sex (59.4%), a mean age at diagnosis of 53.1 years, and has an incidence of 1.63/100,000 [1].

The classic clinical presentation includes progressive dysphagia to both solids and liquids, regurgitation of undigested food, chest discomfort, and weight loss. However, extraesophageal manifestations are increasingly recognized and may predominate in some patients. These manifestations can be respiratory in nature, including chronic cough, wheezing, and dyspnea. Recurrent aspiration pneumonia can also be seen as a consequence of esophageal stasis, leading to the retrograde flow of retained food contents in the esophagus [3,4]. These pulmonary manifestations can obscure the diagnosis, particularly in younger patients, where conditions such as asthma or gastroesophageal reflux disease (GERD) are more commonly initially considered [5].

Delayed recognition carries substantial clinical consequences. Progressive esophageal dilation may result in severe functional obstruction, nutritional compromise, and recurrent pulmonary infections due to chronic aspiration [1,2]. Atypical symptoms are common; more than half of patients report non-classic presentations. These presentations include heartburn, vomiting, or respiratory complaints, which often lead to misdiagnosis and prolonged evaluation prior to definitive diagnosis [5].

Advances in diagnostic modalities, particularly high-resolution manometry, have improved classification of achalasia into clinically meaningful subtypes that inform therapeutic decision-making [2]. Definitive treatments, including pneumatic dilation, laparoscopic Heller myotomy, and peroral endoscopic myotomy (POEM), aim to relieve functional obstruction and reduce the risk of complications such as aspiration-related pulmonary disease [1].

We report a case of a young adult male initially treated for an acute asthma exacerbation whose persistent respiratory symptoms prompted further evaluation, revealing advanced achalasia complicated by aspiration pneumonia. This case highlights the diagnostic challenges posed by extraesophageal presentations and underscores the importance of maintaining a broad differential diagnosis when respiratory and GI symptoms coexist to prevent delayed diagnosis.

## Case presentation

A 20-year-old male with a past medical history of asthma, GERD, gastroparesis, chronic hydrocephalus, and bipolar disorder presented to the emergency department with shortness of breath, productive cough, abdominal pain, and nausea for one day. On physical examination, he exhibited bilateral expiratory wheezing without increased respiratory effort. Given the patient’s history of childhood asthma, he was treated for a suspected asthma exacerbation, and a chest X-ray was ordered. The patient was subsequently treated with nebulized albuterol 0.083%, budesonide 0.25 mg, and ipratropium bromide 0.5 mg; shortness of breath subsided.

The chest X-ray revealed no evidence of acute chest disease but demonstrated tracheal deviation with suspected mediastinal adenopathy (Figure 1). A CT scan of the chest was then ordered to rule out pneumonia and malignancy, although this was considered less likely given his age. At that time, the patient continued to have a productive cough of white sputum with associated nausea. CT of the chest also demonstrated scattered nodular and ground-glass opacities concerning aspiration pneumonia. Additionally, the esophagus was markedly dilated and fluid-filled up to the upper esophageal sphincter, measuring 7.1 cm in the proximal portion and 3.4 cm in the distal portion (Figure 2). The patient was started on IV methylprednisolone and IV cefepime for aspiration pneumonia and was admitted with a diagnosis of acute asthma exacerbation in the setting of achalasia.

**Figure 1:**
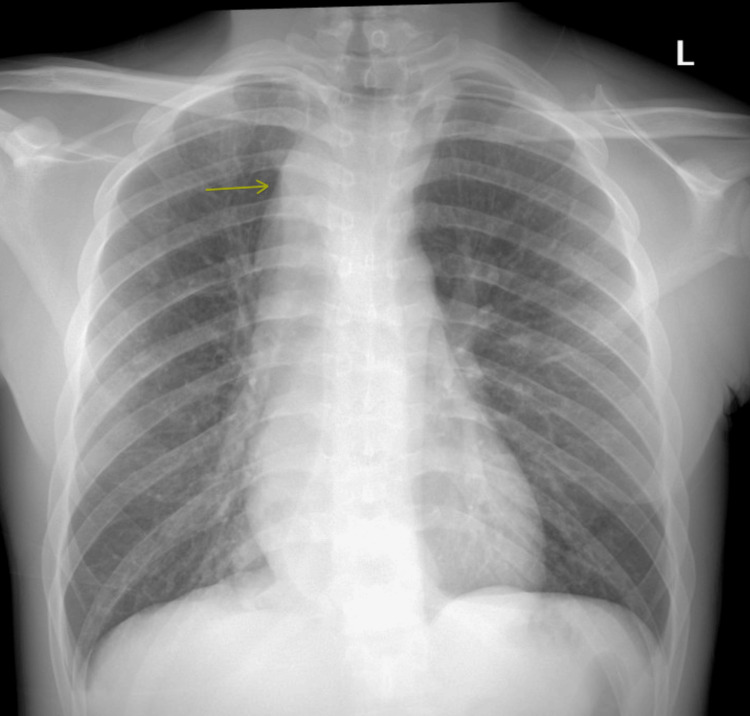
Chest X-ray Abnormal findings include mediastinal prominence and tracheal deviation (arrow) to the right.

**Figure 2:**
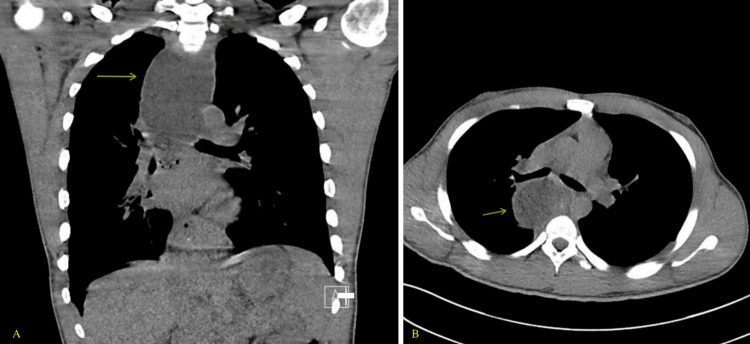
CT chest without contrast: (A) coronal section and (B) axial section Significantly dilated, patulous esophagus (arrows) containing fluid, with fluid contents extending to the level of the upper esophageal sphincter, accounting for the chest X-ray abnormalities. The upper esophagus measures up to 7.1 cm in diameter, and the distal esophagus measures approximately 3.4 cm. The appearance is suggestive of achalasia or pseudoachalasia. Scattered nodular and ground-glass opacities within the right lung and left lower lobe clinically correlate with possible aspiration pneumonia. No mediastinal or hilar adenopathy is appreciated.

At this time, the patient was aware of chronic GERD that was refractory to multiple treatments. Esophagogastroduodenoscopy (EGD) three years prior had demonstrated gastroparesis, and he was being treated with erythromycin 250 mg three times daily; however, the patient had difficulty complying with this medication regimen. An additional EGD was performed, which revealed dilation in the upper and middle third of the esophagus, increasing the suspicion for achalasia (Figure 3). Retained food contents were removed, and a biopsy was taken from the lower third of the esophagus, showing squamous mucosa with basal cell hyperplasia and elongated lamina propria papillae. These cellular changes are consistent with damage due to reflux esophagitis.

**Figure 3:**
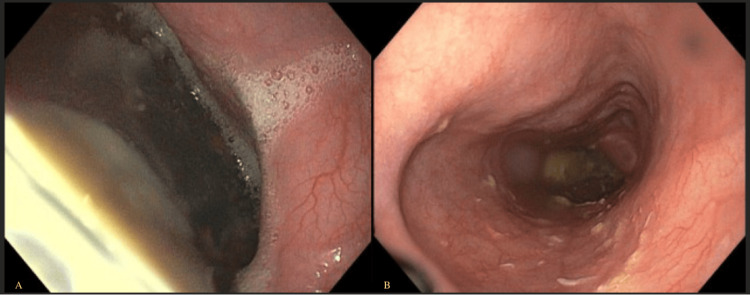
Endoscopy of the upper third (A) and the middle third (B) of the esophagus Dilated upper and middle thirds of the esophagus are seen during endoscopy.

As the patient’s shortness of breath improved, he was transitioned to oral steroids. He was discharged home with albuterol as needed, inhaled corticosteroids, and long-acting bronchodilators and was scheduled for manometry at an outside institution.

## Discussion

This case illustrates an important and underrecognized presentation of achalasia in which respiratory manifestations predominate over classic esophageal symptoms. Although dysphagia and regurgitation remain hallmark features, pulmonary complications are increasingly recognized as a major source of morbidity and may represent the initial presenting complaint [3,4]. The patient described here presented with shortness of breath and a productive cough in the setting of known asthma, resulting in initial treatment directed toward a presumed asthma exacerbation. Similar diagnostic trajectories have been described in prior reports in which respiratory symptoms obscured the underlying esophageal motility disorder, contributing to delayed recognition and advanced disease at diagnosis [5].

Aspiration pneumonia represents one of the most clinically significant extraesophageal complications of achalasia. Esophageal stasis and impaired clearance promote chronic retention of food and secretions, which may be aspirated during recumbency or transient LES relaxation. Imaging findings in this case, including a markedly dilated, fluid-filled esophagus and pulmonary ground-glass opacities, are consistent with previously reported cases of aspiration-related pulmonary disease secondary to advanced achalasia [3,6]. Research indicates that 70% of patients experience reflux, the second most common symptom of achalasia. This condition frequently triggers secondary respiratory issues, including coughing, wheezing, hoarseness, and bronchitis [7,8]. In our case, the patient presented with respiratory symptoms, including shortness of breath, productive cough, wheezing, nausea, abdominal pain, and chest discomfort.

Recent case reports continue to emphasize the heterogeneity of achalasia presentations in young adults and the potential for overlap with atypical systemic findings, reinforcing the importance of multidisciplinary evaluation [9]. For example, atypical symptoms occur in over 60% of patients with achalasia, including heartburn (16.3%), vomiting (15.3%), and belching (7.7%), leading to misdiagnoses of GERD, eosinophilic esophagitis, and other conditions, including psychiatric, neurologic, cardiologic, and thyroid diseases [5]. The degree of esophageal dilation observed is clinically relevant, as increasing esophageal diameter has been associated with more severe disease burden and higher risk of respiratory complications [4].

Comorbid GI conditions in this patient, including GERD and gastroparesis, further contributed to diagnostic complexity. Symptom overlap between achalasia and other esophageal disorders frequently leads to misclassification and delayed definitive evaluation. Studies demonstrate that atypical symptoms such as heartburn and regurgitation are commonly attributed to GERD prior to diagnosis of achalasia, particularly in younger individuals [5,10]. Additionally, emerging evidence suggests associations between achalasia and allergic or atopic conditions, including asthma, which may further confound clinical assessment in patients presenting with respiratory complaints [11,12].

The diagnostic pathway in this case highlights the value of cross-sectional imaging in identifying structural abnormalities when initial evaluation for common respiratory etiologies is unrevealing. CT demonstrated a markedly dilated esophagus extending to the upper esophageal sphincter, prompting endoscopic evaluation and supporting the diagnosis of advanced achalasia. While endoscopy is useful for excluding mechanical obstruction or pseudoachalasia, high-resolution manometry remains the gold standard for diagnosis and subtype classification [1,2].

Therapeutic management of achalasia aims to reduce LES outflow resistance and restore esophageal emptying. Available interventions, including pneumatic dilation, laparoscopic Heller myotomy, and POEM, have demonstrated high efficacy in symptom control and may reduce aspiration risk by improving esophageal clearance [1]. Early recognition is therefore essential to prevent irreversible esophageal dilation and progressive pulmonary complications. In our case, the patient was referred to an outside institution, NYU Langone Health, for a specialized gastroenterology appointment for planned manometry and possible sphincterotomy.

This case reinforces several clinically important principles: respiratory manifestations may represent the initial presentation of achalasia; coexistence of asthma or GERD does not exclude an underlying motility disorder; and persistent or unexplained pulmonary symptoms warrant evaluation for esophageal pathology when supported by clinical context. Early recognition and targeted therapy remain essential to prevent recurrent aspiration, progressive esophageal dysfunction, and long-term morbidity.

## Conclusions

In young patients with recurrent or atypical respiratory symptoms, especially when standard asthma or GERD management fails, achalasia should be considered as a potential underlying cause. Early imaging and endoscopic evaluation are critical to avoid delayed diagnosis, which can lead to aspiration pneumonia and other serious complications. Our case emphasizes the importance of maintaining a broad differential diagnosis when respiratory complaints, such as shortness of breath and productive cough, overlap with GI symptoms such as abdominal pain and nausea. Although less commonly seen, when these symptoms coexist, physicians should remain open to less frequent but clinically significant etiologies, including esophageal motility disorders such as achalasia. Timely recognition of esophageal motility disorders is essential.
